# Yangxinshi Tablet Improves Exercise Capacity for Patients with Coronary Heart Disease: Results from a Randomized, Double-Blind, Placebo-Controlled, and Multicenter Trial

**DOI:** 10.31083/j.rcm2308266

**Published:** 2022-07-21

**Authors:** Sisi Zhang, Yuqin Shen, Peiliang Liu, Xiaoping Meng, Dayi Hu

**Affiliations:** ^1^Division of Cardiology, Department of Internal Medicine, Tongji Hospital, Tongji Medical College of Huazhong University of Science and Technology, 430030 Wuhan, Hubei, China; ^2^Department of Cardiovascular Medicine, Research Center for Translational Medicine, Tongji Hospital Affiliated with Shanghai Tongji University, 200065 Shanghai, China; ^3^Department of Cardiovascular Medicine, The Jinqiu Hospital of Liaoning Province, 110067 Shenyang, Liaoning, China; ^4^Department of Cardiovascular Medicine and Cardiac Rehabilitation Center, Affiliated Hospital of Changchun University of Traditional Chinese Medicine, 130000 Changchun, Jilin, China; ^5^Heart Center, Peking University People's Hospital, 1000044 Beijing, China

**Keywords:** Yangxinshi tablets, coronary heart disease, exercise capacity, anxiety and depression

## Abstract

**Objective:**

To assess the clinical effectiveness of Yangxinshi (YXS) tablets on exercise capacity and symptoms of anxiety and depression in patients with coronary heart disease (CHD).

**Methods and Results:**

A randomized, double-blind, placebo-controlled, multicenter clinical trial was performed to assess the effects of YXS tablets on exercise capacity and quality of life in patients with CHD. A total of 82 patients were included in this trial. Compared with the placebo group, the YXS group showed significant improvement in peak ${\mathrm{VO}}_{2}$ (0.22 L/min vs 0.01 L/min; difference 0.1, 95% confidence interval (CI) 0.04–0.16, *p* = 0.000), peak Mets (0.58 vs 0.09; difference 0.3, 95% CI 0.12–0.47, *p* = 0.005), anaerobic threshold (AT) ${\mathrm{VO}}_{2}$ (0.23 L/min vs 0.04 L/min; difference 0.12, 95% CI 0.07–0.18, *p* = 0.000), AT Mets (0.62 vs 0.16; difference 0.35, 95% CI 0.2–0.5, *p* = 0.001), and 6 minutes walking test (6MWT) (50.05 m vs 11.91 m; difference 29.92, 95% CI 18.78–41.07, *p* = 0.000). There were no differences in Hamilton anxiety rating scale (HAM-A score (1.97 vs 2.07; difference 2.03, 95% CI 0.99–3.06, *p* = 0.926) and Hamilton depression rating scale (HAM-D) score (1.06 vs 1.7; difference1.42, 95% CI 0.24–2.6, *p* = 0.592).

**Conclusions:**

In patients with CHD, YXS tablets, compared with placebo, could improve exercise capacity, without beneficial effects on anxiety and depression symptoms.

## 1. Introduction

Coronary heart disease (CHD) is the leading cause of death worldwide [1]. CHD includes all heart diseases caused by coronary atherosclerosis or spasm, resulting in blood vessel stenosis or obstruction, leading to myocardial ischemia, angina, and myocardial infarction [2]. The traditional treatments recommended by the guidelines include anti-inflammatory, anti-platelet, and lipid-lowering agents, β-receptor blockers, calcium channel blockers, renin-angiotensin system inhibitors, and controlling all risk factors. Although rigorous interventions such as percutaneous coronary intervention (PCI) and coronary artery bypass grafting (CABG) have been implemented in clinical practice, exercise intolerance and other psychological disorders continue to be prevalent in the vast majority of CHD patients [3, 4, 5, 6].

Exercise-based cardiac rehabilitation (CR) significantly improves exercise capacity for patients with CHD, and is considered a Class Ia recommendation by international guidelines [7, 8]. Previous studies had reported that CR is a safe and effective intervention to improve exercise capacity and quality of life in patients with CHD [9, 10, 11]. Improvements in exercise capacity significantly reduce all-cause and cardiovascular mortality by up to 20%–25%. The benefits of reducing cardiovascular risk factors on quality of life have also been established for CHD patients [12, 13]. Despite these well-known benefits, participation in CR programs remains low [14, 15]. Traditional Chinese medicine (TCM) has recently been shown to be advantageous in treating CHD. Hence, integrated Chinese and Western medicine treatments combined with exercise-based CR may improve exercise capacity in patients with CHD.

According to the theory of TCM, Qi deficiency and blood stasis (QDBS) is the most common syndrome in patients with CHD [16]. Many herbal formulas and extracts are used to tonify Qi and nourish Yin in the clinic, such as Astragalus membranaceus Ginseng, and Codonopsis pilosula. Yangxinshi (YXS) tablet, which consists of thirteen kinds of chemical compounds, is widely used to treat patients with CHD and heart failure based on the theory of Reinforcing Qi and Activating Blood [17]. Since limited research is available for TCM in improving exercise capacity for patients with CHD, we conducted a randomized, double-blind, placebo-controlled, multicenter trial to demonstrate whether the YXS tablets can be a suitable adjunct to exercise-based CR for improving exercise capacity and quality of life in patients with CHD.

## 2. Method

The design, criteria, and study procedure has been described in the published protocol (Trial registration:ClinicalTrials.gov with the ID NCT03478332) [18]. Patients were recruited from Three-Grade A-level hospitals in mainland China, including the Affiliated Hospital of Changchun University of Traditional Chinese Medicine, Tongji Hospital Affiliated with Shanghai Tongji University, and Jinqiu Hospital in Liaoning Province. CHD was rated by the Canadian Cardiovascular Society grading of angina pectoris (CSS). In order to obtain a significant difference between groups (a = 0.05, power of 80%), a minimum of 30 patients in each group was needed.

### 2.1 Intervention

All eligible patients signed an informed consent and were then randomly allocated to the intervention group (YXS group) and control group (placebo group) at a ratio of 1:1 with blinding to both patients and investigators. All patients received standard treatment for CHD, including angiotensin-converting enzyme inhibitor (ACEI) or angiotensin receptor blocker (ARB), beta-blockers, calcium antagonistsand, anti-platelet aggregation, and or statins, combined with exercise-based CR in the hospital for 12 weeks. Other similar Chinese patent medicines were prohibited during the trial period. Eligible patients receive three tablets of YXS or placebo (sponsored by Qingdao Growful Pharmaceutical Co.Ltd. Qingdao, Shandong Province, China) three times a day for 12 consecutive weeks.

### 2.2 Exercise-Based CR

Aerobic exercise training included in the CR program was performed three days per week for 12 weeks. Each session lasted for 30 minutes, and all participants were instructed to exercise on a cycle ergometer or treadmill. The target heart rate was obtained by cardiopulmonary exercise testing (CPET). A trained physiotherapist closely supervised the CPET. The training intensity was determined based on the recorded during the CPET examination. Warm-up and cool-down periods were performed in accordance with American College of Sports Medicine guidelines [19]. All the participants were supervised by the CR team consisting of cardiologists, trained physiotherapists, and nurses.

### 2.3 Procedures 

At the first visit, all patients had their medical history, physical examination, concomitant medicine, 12-lead electrocardiography (ECG), CPET, 6 minutes walking test (6MWT), Hamilton anxiety rating scale (HAM-A), and Hamilton depression rating scale (HAM-D) taken. Blood samples were taken for urinalysis, blood count, lipid levels, liver, and renal function. Follow-up visits were conducted every week (with a window of ± 3 days). At the last follow-up visit performed ECG, CPET, 6MWT, HAM-A, HAM-D, and laboratory tests were performed.

### 2.4 Endpoints

#### 2.4.1 Exercise Capacity Testing

CPET and 6MWT evaluated exercise capacity. CPET was performed in accordance with the Exercise Standards for Testing and Training from the American Heart Association [20, 21]. 6MWT was performed in a 30 m corridor, following the guidelines of The American Thoracic Society [22]. The primary expression of exercise capacity is peak oxygen uptake (${\mathrm{VO}}_{2}$), determined by the symptom-limited CPET, which was performed with the Bruce protocol on a bicycle ergometer (Schiller, Switzerland), as previously described. Continuous monitoring with a 12-lead electrocardiogram and breath gas exchange was recorded during the test. Blood pressure was measured every five minutes. Once patients felt exhausted or the monitor showed abnormal signals, the test was ended. Peak ${\mathrm{VO}}_{2}$ was determined as the mean value of ${\mathrm{VO}}_{2}$ observed during the last 30 s of the exercise test. The anaerobic threshold (AT) was calculated using the V-slop method [23]. A metabolic equivalent (MET) is defined as the consumption of oxygen under the resting state of an adult, which is a good predictor of survival in a healthy person [24, 25]. The average MET without exercise is about 3.5 mL/kg/min, with the standing MET rate approaching 4.0 mL/kg/min.

Peak ${\mathrm{VO}}_{2}$ and anaerobic metabolic threshold oxygen consumption (AT-${\mathrm{VO}}_{2}$) were the gold standards to evaluate exercise tolerance [26, 27]. The difference in walking distance (in meters) during 6MWT was also calculated.

#### 2.4.2 Anxiety and Depression 

A 14-item version of the HAM-A and a 17-item version of the HAM-D were used to assess anxious and depressive symptoms, which were commonly used in clinical practice for cardiac patients [28]. Higher HAM-A and HAM-D scores indicate more anxiety and depression.

### 2.5 Safety Assessment

#### 2.5.1 Physical Examination

Patients’ weight and height were measured with a calibrated scale to calculate the body mass index (BMI) value. An automatic sphygmomanometer measured blood pressure and heart rate from the non-dominant arm (J750L, Omron, Osaka, Japan). All participants were instructed to remain at a stabilization time of at least 10 min before the test. We performed the measurement twice and then calculated the mean value as the measurement value.

#### 2.5.2 Adverse Event (AE)

The definition of AE was the appearance or deterioration of any syndrome, symptom, or disease that may affect participants’ health during the trial period. This may be a new disease, degeneration of symptoms, treatment, or a combination of one or more factors. The supervisor carefully recorded all the details, such as manifestation, occurrence time, duration, and degree of AE. Once an AE occurreds, researchers immediately provided optimal medical treatment. Any AE was submitted to the ethics committee within 24 hours.

### 2.6 Statistical Analysis

Data were analyzed by SAS software Version 9.4 (SAS Institute, Cary, NC, USA). Categorical variables were expressed as a percentage, while distributed continuous variables were expressed as mean ± standard error. Differences within the groups were analyzed using χ^2^ and the paired *t*-test, and differences between groups used an independent sample *t*-test. All statistical tests were two-tailed, and *p*-values < 0.05 were considered statistically significant.

## 3. Results

**Fig. 1.**
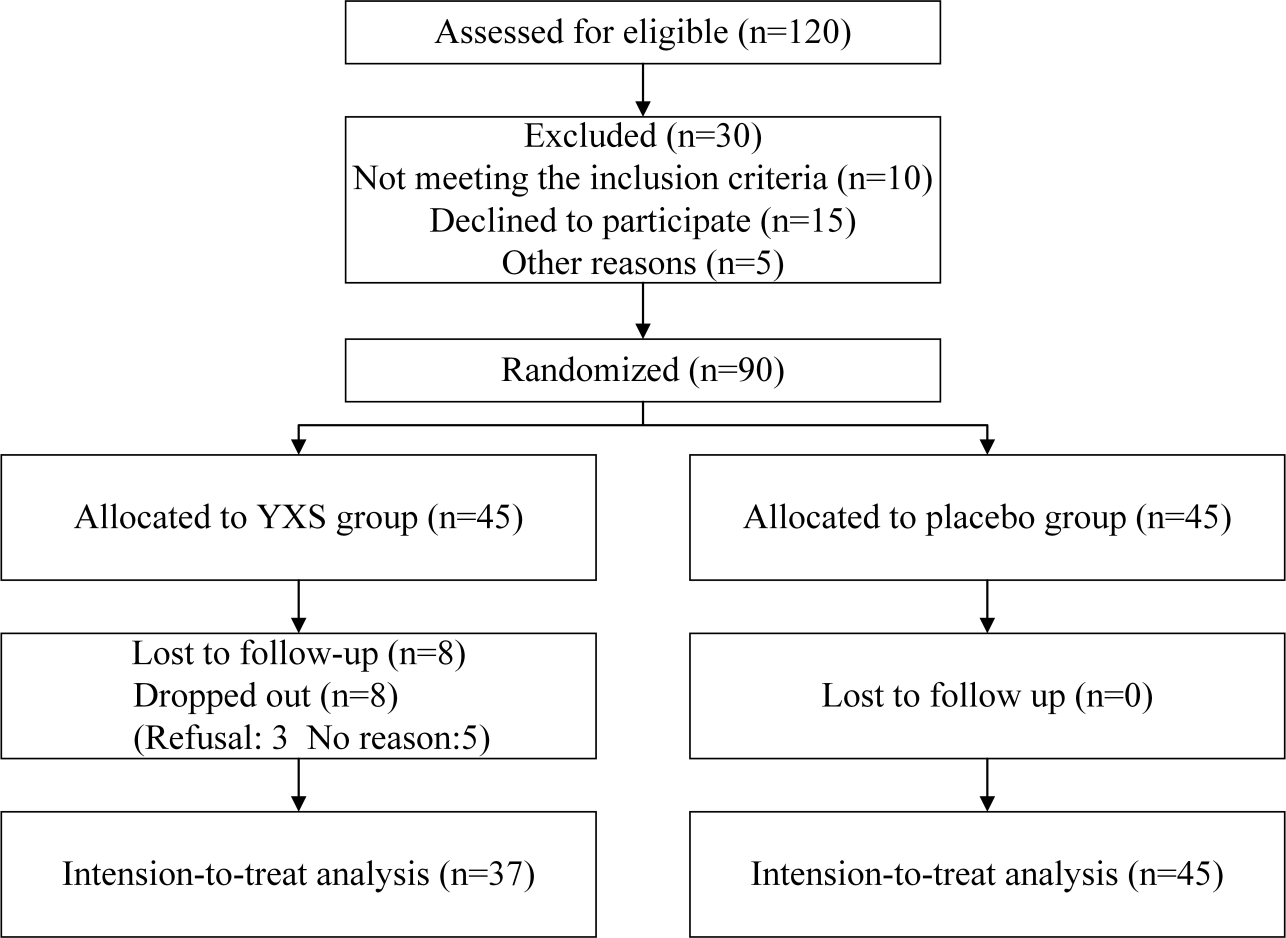
**Flowchart of the study**.

Eighty-two patients were included in the trial. Thirty-seven patients were randomly assigned to the YXS group, and 45 patients were assigned to the control group (Fig. 1). The baseline characteristics of the two groups are shown in Table 1. There was no significant difference between these two groups except for the height.

**Table 1.** **Baseline characteristics**.

		Placebo group (n = 45)	YXS group (n = 37)	t/$\chi^{2}$	*p*-value
Height (cm)		166.51 ± 7.74	172.78 ± 6.28	–4.051	<0.001
Weight (kg)		70.57 ± 9.96	73.72 ± 10.45	–1.394	0.167
BMI (kg/$m^{2}$)		25.39 ± 2.67	24.61 ± 2.66	1.329	0.188
Age, yr		60.20 ± 9.64	59.57 ± 9.54	0.297	0.767
Sex				9.927	0.002
	Male	26 (57.8%)	33 (89.2%)		
	Female	19 (42.2%)	4 (10.8%)		
Angina class				0.122	0.727
	CCS I	30 (66.7%)	26 (70.3%)		
	CCS II	15 (33.3%)	11 (29.7%)		
Therapy, no. (%)					
	Aspirin	40 (88.9%)	35 (94.6%)	0.274	0.601
	Clopidogrel	16 (35.6%)	8 (21.6%)	1.904	0.168
	Statin	42 (93.3%)	34 (91.9%)	0.000	1.000
	Beta adrenergic blockers	28 (62.2%)	22 (59.5%)	0.065	0.799
	Calcium channel blocker	8 (17.8%)	7 (18.9%)	0.018	0.894
	ACE inhibitors (or ARB)	15 (33.3%)	10 (27.0%)	0.381	0.537
	Nitrates	9 (20.0%)	6 (16.2%)	0.194	0.659

BMI, body mass index; ACE, angiotensin converting enzyme; ARB, angiotensin II receptor blocker; CCS, Canadian Cardiovascular Society grading of angina pectoris.

### 3.1 Exercise Capacity

Table 2 presents the exercise capacity results from baseline to 3 months between the groups. Treatment with YXS tablets was associated with a significant improvement in the distance of 6MWT compared to placebo (mean difference 29.92, 95% CI 18.78–41.07, *p *< 0.001).

**Table 2.** **Exercise capacity comparison between the YXS group and the placebo group**.

	Placebo group			*p*-value	YXS group			*p*-value	Changes (95% CI)	between-group *p*-value
	Baseline	3 months	Change		Baseline	3 months	Change			
Distance (m)	470.44 ± 55.49	482.36 ± 54.46	11.91 ± 44.41	0.079	472.19 ± 70.50	522.24 ± 79.96	50.05 ± 50.07	<0.001	29.92 (18.78–41.07)	<0.001
AT ${\mathrm{VO}}_{2}$ (L/min)	0.85 ± 0.24	0.89 ± 0.26	0.04 ± 0.22	0.229	0.90 ± 0.22	1.14 ± 0.38	0.23 ± 0.25	<0.001	0.12 (0.07–0.18)	<0.001
AT MET	3.30 ± 0.62	3.46 ± 0.72	0.16 ± 0.60	0.088	3.34 ± 0.89	3.96 ± 1.30	0.62 ± 0.67	<0.001	0.35 (0.20–0.50)	0.001
${\mathrm{PeakVO}}_{2}$ (L/min)	1.19 ± 0.35	1.19 ± 0.40	0.01 ± 0.18	0.774	1.24 ± 0.31	1.46 ± 0.42	0.22 ± 0.28	<0.001	0.10 (0.04–0.16)	<0.001
Peak MET	4.59 ± 0.99	4.68 ± 1.31	0.09 ± 0.73	0.405	4.67 ± 1.17	5.25 ± 1.37	0.58 ± 0.78	<0.001	0.30 (0.12–0.47)	0.005
Peak WR (w)	94.16 ± 29.76	97.09 ± 31.00	2.93 ± 18.55	0.300	93.28 ± 26.99	105.19 ± 32.80	11.92 ± 11.97	<0.001	6.87 (3.13–10.61)	0.014

AT, anaerobic threshold; MET, metabolic equivalents; WR, work rate.

The YXS-CR combination increased the peak ${\mathrm{VO}}_{2}$ 0.22 ± 0.28 L/min vs placebo 0.01 ± 0.18, with a between-group difference of 0.1 L/min, 95% CI: 0.04–0.16 L/min, *p* = 0.000. Compared with the placebo group, the YXS group significantly increased AT ${\mathrm{VO}}_{2}$ (treatment effect = 0.12 L/min, 95% CI: 0.07–0.18 L/min, between-group *p* = 0.000), AT MET (treatment effect = 0.35, 95% CI: 0.2–0.5, between-group: *p* = 0.001), and peak MET (treatment effect = 0.3, 95% CI: 0.12–0.47, between-group: *p* = 0.005).

### 3.2 Psychological Functioning

There was no significant difference in anxiety and depression scores between the two groups (*p *> 0.001, Table 3). However, compared within groups, both YXS and placebo groups had a significant decrease in HAD-A (Pyxs = 0.007, Pplacebo = 0.012).

**Table 3.** **Anxiety and depression comparison between the YXS group and the placebo group**.

	Placebo group			P1	YXS group			P2	Changes (95% CI)	*p*-value
	Baseline	3 months	Change		Baseline	3 months	change			
HAM-A	6.98 ± 6.58	4.91 ± 4.30	2.07 ± 4.82	0.007	5.63 ± 6.00	3.00 ± 3.57	1.97 ± 4.34	0.012	2.03 (0.99–3.06)	0.926
HAM-D	8.18 ± 8.64	6.48 ± 6.19	1.70 ± 5.91	0.062	6.17 ± 6.52	4.76 ± 5.45	1.06 ± 4.24	0.155	1.42 (0.24–2.60)	0.592

### 3.3 Safety Analysis

There were no patients who had adverse events during the period of the trial. There were no significant ECG or physical examination findings or changes in laboratory parameters associated with the experimental drugs.

## 4. Discussion

This trial was designed to evaluate the effect of adjunctive YXS tablets on exercise-based CR in patients with CHD and the underlying mechanisms of TCM in improving exercise capacity.

Our findings demonstrated that YXS tablets significantly increased exercise capacity in patients with CHD.

Previous studies have shown that mitochondrial biogenesis and mitochondrial adenosine triphosphate (ATP) production supply energy for cellular biosynthesis, and mitochondrial dysfunction is a characteristic of exercise intolerance [29, 30, 31]. As a traditional patent Chinese medicine, YXS tables consist of thirteen compounds: Astragalus membranaceus, Codonopsis pilosula, Salvia miltiorrhiza, Pueraria, epimedium, hawthorn, Rehmannia glutinosa, angelica, Coptis Chinensis, corydalis, Ganoderma lucidum, ginseng, and licorice. A network pharmacology-based study has shown that YXS tables could increase myocardial mitochondrial membrane potential, expiratory chain I activity, and ATP levels in rat models, thereby promoting mitochondrial biogenesis and aerobic metabolism. Previous animal studies also showed that YXS tablets improved energy metabolism in rats, thus increasing exercise tolerance [32]. Other studies have confirmed its antioxidant and anti-depression properties [33, 34].

According to the theory of TCM, Qi, which includes oxygen and nutrition, flows through the whole body, promotes blood circulation, and induces ATP synthase in the mitochondria [35]. Qi deficiency may lead to blood stasis, eventually leading to a decline in exercise tolerance. From the perspective of TCM, the primary mechanism of improving exercise capacity lies in regulating Qi, activating blood, and then improving mitochondrial energy metabolism. Previous investigations have demonstrated some tonic herbs with the effects of Qi-invigorating and increasing mitochondrial ATP generation, which may enhance exercise capacity [36]. YXS tablets, Ginseng, Astragalus membranaceus, Coptis Chinensis, and puerarin are all related to modulating energy metabolism. Moreover, salvia miltiorrhiza could also regulate energy metabolism via the phosphorylated-Jun N-terminal kinase-kappaB transient receptor potential cation channel, subfamily C, member 6 (p-JNK-NF-kappaB-TRPC6) pathway, and Rho kinase (ROCK) dependent ATP5D modulation [37, 38, 39].

It is well known that (GLU) is the primary energy source for the heart; GLUT4 transports glucose to the mitochondria for utilization [40]. Ginseng and Salvia miltiorrhiza can increase glucose uptake via the GLUT4 and Adenosine 5’monophosphate-activated protein kinase (AMPK) signaling pathways *in vivo * [41, 42]. The active compound of Coptis Chinensis, Berberine can moderate glucose metabolism via the AMPK/peroxisome proliferator-activated receptor coactivator (PGC)-1ɑ/GLUT4 pathway [43]. The compounds in the YXS tablets are involved in glucose metabolism, which suggests that this is a potential therapeutic strategy for exercise intolerance. Multiple mechanisms contribute to the benefits of the YXS tablets in improving exercise capacity. A significant difference in peak ${\mathrm{VO}}_{2}$ (YXS group: 0.22 L/min vs placebo group: 0.01 L/min, *p* = 0.000) was found in our study at 12 weeks; in addition, the YXS group increased the walking distance by about 50 m, which is a significant improvement [44]. This study has demonstrated a positive effect on exercise capacity. Increased peak ${\mathrm{VO}}_{2}$ could delay disease progression and improve disease prognosis for patients with CHD. As reported in a previous study, the improvements in peak ${\mathrm{VO}}_{2}$ after 12-week CR programs are around 10–20% and significantly impact mortality and CV prognosis. These results are clinically meaningful, as it reported that 1/kg/min improvement in ${\mathrm{VO}}_{2}$ peak during a CR program had been associated with a 6% reduction in hospital readmissions and a 13% reduction in all-cause mortality [45, 46].

However, no difference was found in anxiety and depression between groups, indicating that the improvement of exercise capacity by YXS tablets might not be necessarily related to an improvement in psychological parameters. There are several possible reasons for this. First, the population included in both the YXS and placebo groups had lower baseline HAM-A (5.63 vs 6.98) and HAM-D (6.17 vs 8.18) scores, indicating no/minimal anxiety and depression. Second, our trial was only conducted for three months, which may not be a sufficient time to notice improvements in anxiety and depression. Additionally, the lack of psychological rehabilitation intervention, including patient education, may contribute to these outcomes during the intervention period.

This trial demonstrates the unique advantages of TCM in improving exercise capacity and provides the theoretical basis for herbal medicine in rehabilitating patients with CHD. The CR program has studied many methods from TCM to improve exercise capacities, such as Taiji and Ba Duanjin. We and others have found that YXS tablets significantly improved exercise capacity in patients with CHD. In our study, CR was delivered via a supervised center-based program. Due to the advances in telemedicine technology, home-based cardiac rehabilitation could be seen as a safe and suitable alternative to center-based CR in patients, especially for the elderly, disabled, and patients in rural areas [47]. Based on the results of our study, we propose incorporating YXS tablets and home-based CR as a potential alternative to improve exercise capacity in patients with CHD.

The moderate sample size and the single ethnic patient group are recognized as limitations of this study and will need to be verified in larger studies enlisting multiple ethnic populations.

## 5. Conclusions

Compared with placebo, adding YXS tablets to exercise-based cardiac rehabilitation programs could improve exercise capacity in patients with CHD. However, there were no improvements in anxiety and depression. Further larger studies with longer follow-up and multiethnic patient populations are necessary to further assess the benefits of YXS tablets to improve exercise capacity in patients with CHD.

## 6. Limitation

The limitations of this study include the differences between groups at the baseline, such as sex and height. A randomized controlled trial with a larger sample size could help eliminate such biases in the future.

## References

[1] Lozano R, Naghavi M, Foreman K, Lim S, Shibuya K, Aboyans V, Abraham J (2012). Global and regional mortality from 235 causes of death for 20 age groups in 1990 and 2010: a systematic analysis for the Global Burden of Disease Study 2010. *Lancet*.

[2] Wang X, Song D, Tao T, He T, Wu X, Li X (2021). Qi-Regulating and Blood Circulation-Promoting Therapy Improves Health Status of Stable Angina Pectoris Patients with Depressive Symptoms. *Evidence-Based Complementary and Alternative Medicine*.

[3] Richards SH, Anderson L, Jenkinson CE, Whalley B, Rees K, Davies P (2017). Psychological interventions for coronary heart disease. *Cochrane Library: Cochrane Reviews*.

[4] Alam M, Huang HD, Shahzad SA, Kar B, Virani SS, Rogers PA (2013). Percutaneous Coronary Intervention vs. Coronary Artery Bypass Graft Surgery for Unprotected Left Main Coronary Artery Disease in the Drug-Eluting Stents Era. *Circulation Journal*.

[5] Sipahi I, Akay MH, Dagdelen S, Blitz A, Alhan C (2014). Coronary Artery Bypass Grafting vs Percutaneous Coronary Intervention and Long-term Mortality and Morbidity in Multivessel Disease. *JAMA Internal Medicine*.

[6] Rosendorff C, Lackland DT, Allison M, Aronow WS, Black HR, Blumenthal RS (2015). Treatment of hypertension in patients with coronary artery disease: a scientific statement from the American Heart Association, American College of Cardiology, and American Society of Hypertension. *Hypertension*.

[7] Piepoli MF, Hoes AW, Agewall S, Albus C, Brotons C, Authors/Task Force Members (2016). 2016 European Guidelines on cardiovascular disease prevention in clinical practice: The Sixth Joint Task Force of the European Society of Cardiology and Other Societies on Cardiovascular Disease Prevention in Clinical Practice (constituted by representatives of 10 societies and by invited experts): Developed with the special contribution of the European Association for Cardiovascular Prevention & Rehabilitation (EACPR). *European Journal of Preventive Cardiology*.

[8] Balady GJ, Williams MA, Ades PA, Bittner V, Comoss P, Foody JM (2007). Core Components of Cardiac Rehabilitation/Secondary Prevention Programs: 2007 Update. *Circulation*.

[9] Taylor RS, Brown A, Ebrahim S, Jolliffe J, Noorani H, Rees K (2004). Exercise-based rehabilitation for patients with coronary heart disease: systematic review and meta-analysis of randomized controlled trials. *The American Journal of Medicine*.

[10] Vanhees L, Rauch B, Piepoli M, van Buuren F, Takken T, Börjesson M (2012). Importance of characteristics and modalities of physical activity and exercise in the management of cardiovascular health in individuals with cardiovascular disease (Part III). *European Journal of Preventive Cardiology*.

[11] Mezzani A, Hamm LF, Jones AM, McBride PE, Moholdt T, Stone JA (2013). Aerobic exercise intensity assessment and prescription in cardiac rehabilitation: a joint position statement of the European Association for Cardiovascular Prevention and Rehabilitation, the American Association of Cardiovascular and Pulmonary Rehabilitation and the Canadian Association of Cardiac Rehabilitation. *European Journal of Preventive Cardiology*.

[12] Winnige P, Vysoky R, Dosbaba F, Batalik L (2021). Cardiac rehabilitation and its essential role in the secondary prevention of cardiovascular diseases. *World Journal of Clinical Cases*.

[13] Shepherd CW, While AE (2012). Cardiac rehabilitation and quality of life: a systematic review. *International Journal of Nursing Studies*.

[14] van Engen-Verheul M, de Vries H, Kemps H, Kraaijenhagen R, de Keizer N, Peek N (2013). Cardiac rehabilitation uptake and its determinants in the Netherlands. *European Journal of Preventive Cardiology*.

[15] Turk-Adawi KI, Oldridge NB, Tarima SS, Stason WB, Shepard DS (2014). Cardiac Rehabilitation Enrollment among Referred Patients. *Journal of Cardiopulmonary Rehabilitation and Prevention*.

[16] Wang J, Chu F, Li J, Yao K, Zhong J, Zhou K (2008). Study on syndrome element characteristics and its correlation with coronary angiography in 324 patients with coronary heart disease. *Chinese Journal of Integrative Medicine*.

[17] Zhang C, Mi ZY, Tian WJ (2014). Clinical Observation on Therapeutic Effect of Clopidogrel Bisulfate Tablets Combined with Atorvastatin Calcium Capsules and Yangxinshi Tablets on 78 Cases of Angina Pectoris after PCI. *World Chinese Medicine*.

[18] Zhang S, Liang C, Yang Y, Zhao Z, Li J, Meng X (2020). Effects of Yangxinshi tablet on exercise tolerance in patients with coronary heart disease. *Medicine*.

[19] American College of Sports Medicine Position Stand (1998). The recommended quantity and quality of exercise for developing and maintaining cardiorespiratory and muscular fitness, and flexibility in healthy adults. *Medicine & Science in Sports & Exercise*.

[20] Fletcher GF, Balady GJ, Amsterdam EA, Chaitman B, Eckel R, Fleg J (2001). Exercise Standards for Testing and Training. *Circulation*.

[21] Fletcher GF, Ades PA, Kligfield P, Arena R, Balady GJ, Bittner VA (2013). Exercise Standards for Testing and Training. *Circulation*.

[22] ATS Committee on Proficiency Standards for Clinical Pulmonary Function Laboratories (2002). ATS statement: guidelines for the six-minute walk test. *American Journal of Respiratory and Critical Care Medicine*.

[23] Beaver WL, Wasserman K, Whipp BJ (1986). A new method for detecting anaerobic threshold by gas exchange. *Journal of Applied Physiology*.

[24] Morris CK, Myers J, Froelicher VF, Kawaguchi T, Ueshima K, Hideg A (1993). Nomogram based on metabolic equivalents and age for assessing aerobic exercise capacity in men. *Journal of the American College of Cardiology*.

[25] Gulati M, Black HR, Shaw LJ, Arnsdorf MF, Merz CNB, Lauer MS (2005). The Prognostic Value of a Nomogram for Exercise Capacity in Women. *New England Journal of Medicine*.

[26] Yancy CW, Jessup M, Bozkurt B, Butler J, Casey DE, Drazner MH (2013). 2013 ACCF/AHA guideline for the management of heart failure: a report of the American College of Cardiology Foundation/American Heart Association Task Force on Practice Guidelines. *Journal of the American College of Cardiology*.

[27] Huan N, Wang CL, Wang PL, Yu YH (2021). Advantage evaluation of cardiopulmonary exercise test in clinical rehabilitation of cardiovascular disease. *Chinese Heart Journal*.

[28] Zigmond AS, Snaith RP (1983). The Hospital Anxiety and Depression Scale. *Acta Psychiatrica Scandinavica*.

[29] Ventura-Clapier R, Garnier A, Veksler V (2004). Energy metabolism in heart failure. *The Journal of Physiology*.

[30] Brown DA, Perry JB, Allen ME, Sabbah HN, Stauffer BL, Shaikh SR (2017). Mitochondrial function as a therapeutic target in heart failure. *Nature Reviews Cardiology*.

[31] Rosca MG, Hoppel CL (2013). Mitochondrial dysfunction in heart failure. *Heart Failure Reviews*.

[32] Wu R, Jiang B, Li H, Dang W, Bao W, Li H (2020). A network pharmacology approach to discover action mechanisms of Yangxinshi Tablet for improving energy metabolism in chronic ischemic heart failure. *Journal of Ethnopharmacology*.

[33] Zhu J, Yi X, Zhang J, Chen S, Wu Y (2017). Chemical profiling and antioxidant evaluation of Yangxinshi Tablet by HPLC–ESI-Q-TOF-MS/MS combined with DPPH assay. *Journal of Chromatography B*.

[34] Han J, Li Q, Ma Z, Fan J (2017). Effects and mechanisms of compound Chinese medicine and major ingredients on microcirculatory dysfunction and organ injury induced by ischemia/reperfusion. *Pharmacology & Therapeutics*.

[35] Leong PK, Wong HS, Chen J, Ko KM (2015). Yang/Qi Invigoration: an Herbal Therapy for Chronic Fatigue Syndrome with Yang Deficiency. *Evidence-Based Complementary and Alternative Medicine*.

[36] Ko K-M, Leon TYY, Mak DHF, Chiu P-Y, Du Y, Poon MKT (2006). A characteristic pharmacological action of ‘Yang-invigorating’ Chinese tonifying herbs: Enhancement of myocardial ATP-generation capacity. *Phytomedicine*.

[37] MENG Y, LI W, SHI Y, ZHOU B, MA R, LI W (2016). Danshensu protects against ischemia/reperfusion injury and inhibits the apoptosis of H9c2 cells by reducing the calcium overload through the p-JNK-NF-κB-TRPC6 pathway. *International Journal of Molecular Medicine*.

[38] He K, Yan L, Pan C, Liu Y, Cui Y, Hu B (2014). ROCK-dependent ATP5D modulation contributes to the protection of notoginsenoside NR1 against ischemia-reperfusion-induced myocardial injury. *American Journal of Physiology-Heart and Circulatory Physiology*.

[39] Chen GL, Xu S, Wu ZJ, Wu Y (2020). Protective Effect of Salvianolic Acid B on Intestinal Ischemia-reperfusion Injury in Rats. *Zhongguo Yi Xue Ke Xue Yuan Xue Bao*.

[40] Luiken JJ, Ouwens DM, Habets DD, van der Zon GC, Coumans WA, Schwenk RW (2009). Permissive action of protein kinase C-zeta in insulin-induced CD36- and GLUT4 translocation in cardiac myocytes. *Journal of Endocrinology*.

[41] Wei B, You M, Ling J, Wei L, Wang K, Li W (2013). Regulation of antioxidant system, lipids and fatty acid β-oxidation contributes to the cardioprotective effect of sodium tanshinone IIA sulphonate in isoproterenol-induced myocardial infarction in rats. *Atherosclerosis*.

[42] Zheng X, Wang S, Zou X, Jing Y, Yang R, Li S (2017). Ginsenoside Rb1 improves cardiac function and remodeling in heart failure. *Experimental Animals*.

[43] Zhang Q, Xiao X, Feng K, Wang T, Li W, Yuan T (2011). Berberine Moderates Glucose and Lipid Metabolism through Multipathway Mechanism. *Evidence-Based Complementary and Alternative Medicine*.

[44] Rasekaba T, Lee AL, Naughton MT, Williams TJ, Holland AE (2009). The six-minute walk test: a useful metric for the cardiopulmonary patient. *Internal Medicine Journal*.

[45] Mikkelsen N, Cadarso-Suárez C, Lado-Baleato O, Díaz-Louzao C, Gil CP, Reeh J (2020). Improvement in VO2peak predicts readmissions for cardiovascular disease and mortality in patients undergoing cardiac rehabilitation. *European Journal of Preventive Cardiology*.

[46] Abu-Haniyeh A, Shah NP, Wu Y, Cho L, Ahmed HM (2018). Predictors of cardiorespiratory fitness improvement in phase II cardiac rehabilitation. *Clinical Cardiology*.

[47] Stefanakis M, Batalik L, Antoniou V, Pepera G (2022). Safety of home-based cardiac rehabilitation: A systematic review. *Heart Lung*.

